# Radiomics of portal-phase ring enhancement: a novel imaging biomarker for bevacizumab response associated with overall survival rates. It might help with surgical decision-making in colorectal liver metastases?

**DOI:** 10.1007/s13304-025-02501-w

**Published:** 2026-01-28

**Authors:** Maria Chiara Brunese, Alfredo Clemente, Marco De Chiara, Valerio Nardone, Salvatore Spiezia, Pasquale Avella, Erika Martinelli, Maria Giovanna Chini, Fabrizio Urraro, Alfonso Reginelli, Salvatore Cappabianca, ROI Segmentation Collaborative Group, Paolo Bianco, Francesco Stanzione, Mario Brunese, Anna Russo

**Affiliations:** 1 Department of Medicine and Health Sciences “V. Tiberio”, University of Molise, 86100 Campobasso, Italy; 2 Radiology and Radiotherapy Unit, Department of Precision Medicine, University of Campania “L. Vanvitelli”, Piazza Miraglia 2, 80138 Naples, Italy; 3 Department of Life Sciences, Health and Health Professions, Link Campus University, 00165 Rome, Italy; 4 Hepatobiliary and Pancreatic Surgery Unit, Pineta Grande Hospital, 81030 Castel Volturno, Caserta, Italy; 5 Medical Oncology Unit, Department of Precision Medicine, University of Campania Luigi Vanvitelli, 80131 Naples, Italy; 6 General Surgery Unit, Pineta Grande Hospital, 81030 Castel Volturno, Caserta, Italy

**Keywords:** Colorectal liver metastases (CRLM), Radiomics, Portal phase ring enhancement, Bevacizumab-based chemotherapy, Prognostic imaging biomarkers

## Abstract

The liver is the most common site of metastases from colorectal cancer (CRC), affecting up to half of patients throughout their disease course. Although contrast-enhanced computed tomography (CECT) is routinely used for staging and treatment monitoring, RECIST criteria poorly reflect biological heterogeneity and antiangiogenic therapy effects. Integrating radiological and radiomic biomarkers may enhance response evaluation and personalized treatment. This study aimed to evaluate portal-phase ring enhancement as a potential imaging biomarker of disease progression and prognosis in patients with colorectal liver metastases (CRLM) treated with bevacizumab-based chemotherapy and to explore its correlation with CT-derived radiomic features. Eighty consecutive patients with histologically confirmed CRLM treated with standard chemotherapy plus bevacizumab were retrospectively analyzed. Baseline and 3-month CECT scans were evaluated for the presence and evolution of portal-phase ring enhancement. Radiomic features were extracted and correlated with morphologic patterns, while survival outcomes were assessed using Kaplan–Meier and logistic regression analyses. Baseline portal-phase ring enhancement was observed in 32.5% of patients and was significantly associated with inferior overall survival (*p* = 0.001), a finding confirmed on follow-up imaging (*p* = 0.016). Among radiomic features, *sphericity* showed the strongest correlation with ring enhancement (*p* = 0.003), yielding a modestly discriminative model. Portal-phase ring enhancement represents a reproducible imaging biomarker of poor prognosis in bevacizumab-treated CRLM. Its correlation with distinct radiomic signatures reinforces its biological plausibility as a marker of tumor aggressiveness. Integrating this feature with shape-based metrics into early imaging evaluation may refine risk stratification and personalized management.

## Introduction

Colorectal cancer (CRC) is the third most common tumor worldwide and the second leading cause of cancer-related deaths [1, 2].

Advances in long-term survival outcomes may be ascribed to the progressive integration of multidisciplinary strategies in the management of advanced and metastatic disease [3].

The liver is the most common site of metastasis, accounting for approximately 25% of all metastatic sites across cancers [2, 4, 5]. Liver involvement is present at diagnosis in ~ 35–55% of colorectal cancers and constitutes a key prognostic marker that materially guides therapeutic decision-making [6]. Despite advances in chemotherapy regimens and local treatment surgical resections is still considered the treatment of choice in patients affected by Colorectal liver metastasis (CRLM) [7, 8].

However, the optimal timing for liver resection is still debated, especially considering that even more patients affected by cancer are frail showing a high surgical risk due to multiple comorbidities and pharmacological therapies [9–11].

Contrast-enhanced computed tomography (CE-CT) is the gold standard for evaluating CRLM [12]. However, liver metastases exhibit heterogeneous appearances on CT, and multiple clinical studies have interrogated specific imaging features to predict biological aggressiveness and clinical behavior [13].

CT-based assessment of tumor burden is primarily defined by size metrics per the Response Evaluation Criteria in Solid Tumors (RECIST) [14], which continue to represent the best predictor of prognosis and the most reliable framework for multidisciplinary management.

Although volumetric assessment remains foundational, computed tomography (CT) conveys clinically relevant information beyond lesion size [15]. Advances in computational image analysis now enable high-throughput extraction of quantitative features from CT, establishing a modern paradigm in oncologic imaging, particularly for liver cancer evaluation [16–18]. Radiomics typically quantifies shape, intensity, volume, and texture, capturing aspects of the tumor microenvironment [19]. Integrated with clinical data, these imaging biomarkers can inform histologic characterization, predict therapeutic response, and monitor disease progression across clinical settings [19].

Accurate diagnosis, staging, and management of hepatic metastases materially influence prognosis in CRC [20]. Although radiomic studies in CRLM and chemotherapy response prediction are increasingly reported [21], quantitative correlations of response to specific regimens such as bevacizumab are limited.

Bevacizumab-based chemotherapy improves long-term outcomes and is recommended by the NCCN as first-line therapy [22]. A predictive imaging biomarker for antiangiogenic therapy is therefore needed, yet early response assessment varies widely among patients [23]. Few CT studies during bevacizumab therapy have moved beyond qualitative markers such as perilesional rim enhancement [24]. Portal-phase rim enhancement on CE-CT (peripheral enhancing rim with a relatively hypodense core) is associated with angiogenesis, necrosis, and vascular remodeling in CRLM [25, 26].

Importantly, surgical candidacy for hepatic resection is a dynamic construct that evolves with the biological response to systemic therapy [27]. In patients receiving bevacizumab-based chemotherapy, early morphologic and radiomic indicators of treatment efficacy should prompt systematic re-appraisal of resectability; conversion to surgery may be justified once technical feasibility and oncologic stability are achieved, even in the absence of substantial dimensional shrinkage [5, 7, 28]. Conversely, non-response or unfavorable morphologic evolution ought to temper operative intent and may redirect patients toward continued systemic therapy or other alternatives following multidisciplinary review [27]. Accordingly, imaging-anchored response assessment is integral to individualizing both the timing and the appropriateness of operative intervention.

Our pilot study purposed to quantify portal-phase rim enhancement and the correlation with CT-derived radiomic features to assess its value for predicting disease progression in CRLM patients. This approach may lead to a more accurate definition of patient specific characteristics to better address surgical indication, concerning timing of resection and eventual sparing of unnecessary chemotherapy regimens.

## Materials and methods

### Study design

All consecutive patients affected by CRLM and discussed at multidisciplinary team of the University of Campania “L. Vanvitelli”, Naples (Italy), from August 2018 to February 2020, were included.

This is a retrospective analysis and was performed according to STROBE guidelines [29].

All patients were treated with standard chemotherapy (oxaliplatin [i.e., XELOX or FOLFOX] or irinotecan [i.e., FOLFIRI or XELIRI]) and bevacizumab according to National Guidelines [30].

The inclusion criteria were: age ≥ 18 years, pathologically proven CRC, evidence of CRLM at baseline CT scan and at restaging CT after 3 months from the beginning of bevacizumab-based therapy, ≥ 1 CRLM.

Exclusion criteria included absence/lack of bevacizumab or first-line chemotherapy, incomplete imaging (missing baseline or 3-month follow-up CT), lack of imaging quality, patients affected by other liver neoplasms.

All the patients provided written consent to the anonymous use of their scan images for research purposes.

This study was approved by the Institutional Review Board of the University of Campania “L. Vanvitelli”, Naples, Italy (protocol number 39988, approved date: October 03, 2023).

This study was conducted according to the Declaration of Helsinki.

### CT imaging

All patients underwent a multiphasic CT examination using a 64-slice Revolution EVO scanner (GE Healthcare, Milwaukee, WI, USA), employing a standardized abdomen and pelvis imaging protocol at time of diagnosis and after 3-month from the beginning of chemotherapy regimen.

The protocol included:

- Quadri-phasic acquisition protocol with after intravenous injection of iodinate contrast medium (Iomeron 400, Bracco, Milan, Italy) at the standard dose of 1.5 mL/kg with a flow rate of 3.5 mL/s.
- Collimation thickness 0.625 mm, layer thickness 2.5 mm, tube current 630 mA, rotation speed 0.5 s.

According to RECIST 1.1 criteria [14] liver metastasis larger than 10 mm were considered as **target lesions**.

Lesion progression (LP) was evaluated following the criteria depicted above:

Complete Response (CR): Disappearance of all lesions and pathologic lymph nodes.

Partial Response (PR): ≥ 30% decrease Sum of the Longest Diameters (SLD), no new lesions, no progression of non-target lesions.

Stable Disease (SD): no PR—no PD.

Progression Disease (PD): ≥ 20% increase SLD compared to smallest SLD in study or progression of non-target lesions or new lesions.

### Feature extraction and texture analysis

Volume of Interest (VOI) was manually delineated at both baseline and follow-up by two blinded radiologists with 10 years of experience in oncologic imaging, inter-observer agreement statistics for ring-enhancement was assessed.

Segmentations were performed on both non-contrast and portal venous phase images.

Texture analysis parameters were evaluated for reliability using the Intraclass Correlation Coefficient (ICC) method. Feature extraction and segmentation were conducted using LifeX software (Version 7.2, LITO 22, developed by C. Nioche, INSERM, Paris, France)[31].

An ICC value > 0.75 was considered indicative of good agreement among the segmentations. In cases where the ICC was below this threshold, discrepancies between the two radiologists were solved by a third radiologist with a 30-year experience in oncological imaging.

The extracted texture parameters included:

- Gray-Level Co-Occurrence Matrix (GLCM) features
- Shape descriptors
- Gray-Level Histogram indices

### Endpoints and statistical analysis

By quantifying portal-phase rim enhancement and mapping its relationships to CT-derived radiomic features across multiple domains (morphology, intensity, and texture), we seek to evaluate the robustness and clinical utility of this marker as a predictor of disease progression and Overall Survival (OS) in colorectal liver metastases, thereby informing risk stratification and treatment planning.

**Table 1:** Baseline characteristics of patients enrolled in first analysis

Baseline characteristics	
Sex	39 female and 41 male
Age (years)	Mean 67,18 ±11,26
Number of lesions	Mean 7,28 ±11,60
Mean axial diameter target lesions (mm)	41,05 ±27,89

**Table 2:** Kaplan–Meier analysis associating ring enhancement and overall survival at T0

Case processing summary				
Ring enhancement baseline	Total N.	N. of events	Censored	
			N.	Percent (%)
No	54	54	0	0,0%
yes	26	26	0	0,0%
Overall	80	8O	0	0,0%

Means and medians for survival time								
Ring enhancement baseline	Mean^a^					Median		
	Estimate	Std. Error	95% Confidence Interval		Estimate	Std. Error	95% Confidence Interval	
			Lower Bound	Upper Bound			Lower Bound	Upper Bound
No	15,511	,869	13,808	17,214	18,020	,507	17,026	19,014
Yes	11.875	,927	10,058	13,692	11,530	,293	10,953	12,104
Overall	14,329	,683	12,990	15,663	15,360	1,723	11,982	18.738

Overall comparisons			
	Chi-Square	df	Sig
Log Rank (Mantel-Cox)	10,252	1	,001

a. Estimation is limited to the largest survival time it it is censored

Test of equality of survival distributions for the different levels of ring.enhanc.baseline.

The clinical characteristics of our patient’s data were tested with Chi-Square analysis.

Kaplan–Meier survival curves were generated for survival analysis evaluating our multidisciplinary data. Pearson’s correlation coefficient was applied to identify relevant features and eliminate redundancy. T-tests were used to select significant features for binary logistic regression model development.

All statistical analyses were performed using SPSS software (Version 23.0, IBM Corp., Armonk, NY, USA).

Results were considered statistically significant at a two-sided *P* < 0.05.

## Results

**Fig. 1:**
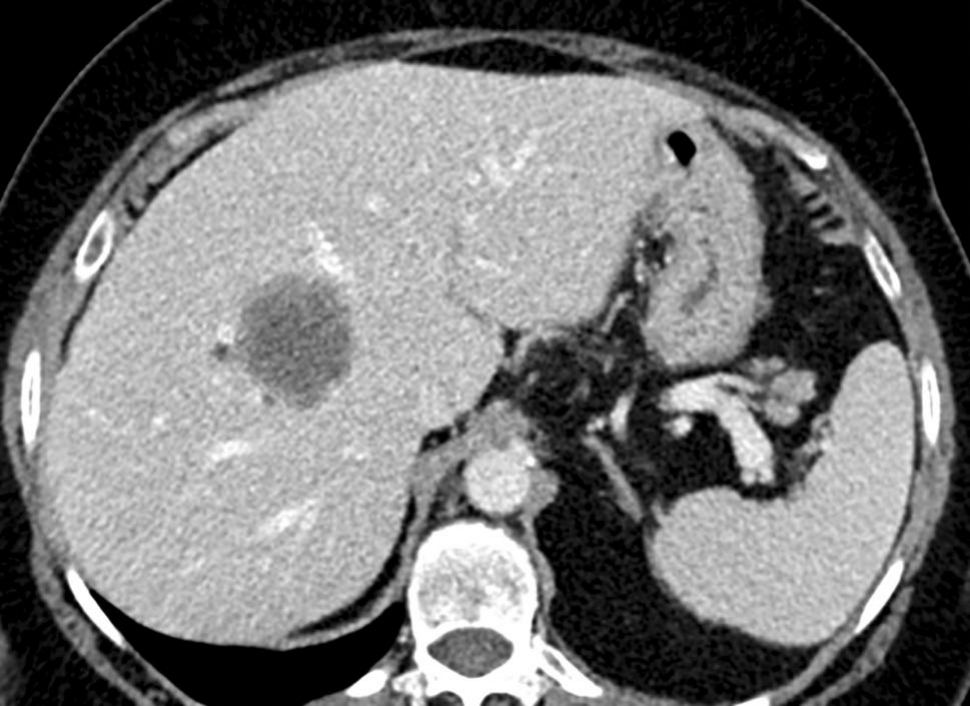
Portal phase of a Computed Tomography scan representing the absence of portal rim enhancement around a liver metastases from colorectal cancer

**Fig. 2:**
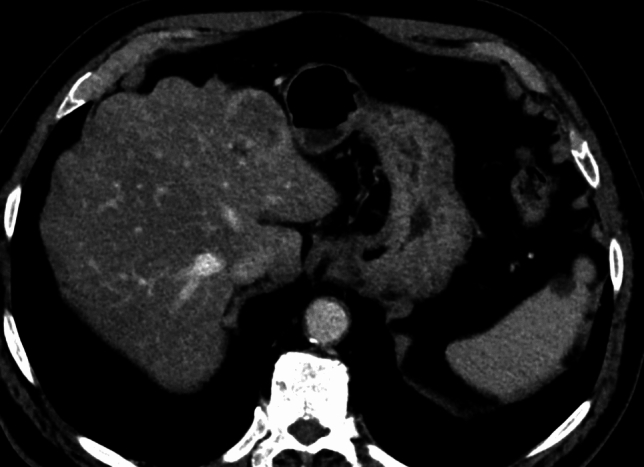
Portal phase Computed Tomography scan representing a liver metastasis surrounded by rim enhancement

Between August 1, 2018, and February 28, 2020, we retrospectively enrolled 80 eligible patients with at least one hepatic metastatic lesion on baseline contrast-enhanced CT. Baseline characteristics are summarized in Table 1. The evaluation was unmasked. On portal-venous–phase imaging at baseline (T0), 54 of 80 patients (67.5%) demonstrated no rim (ring) enhancement, whereas 26 of 80 (32.5%) exhibited rim enhancement. Kaplan–Meier analysis showed that ring enhancement in the portal phase measured at baseline CT was significantly related to overall survival (*p* = *0.001*) (Table 2). In particular, the presence of ring enhancement at baseline CT was related to a worse prognosis (Figs. 1 and 2).

**Table 3:** RECIST evaluation of the target lesions at T1

Patients re-evaluation at first follow-up 3 months	
Target lesions: 81 of 68 patients	
Target lesions increased > 20%	15 target 1 + 11 target 2
Stable lesions	14 target 1 + 23 target 2
Reduced > 30%	13 target 1 + 5 target 2

Within the subsequent 3 months **(T1)**, 12 patients (15.0%) were died or did not perform the FU CT at our center; accordingly, 68 of 80 (85.0%) completed the first scheduled follow-up assessment.

The 3-month follow up (T1) CT scan showed 38 patients (58.9%) with no ring enhancement, while 30 patients (41.1%) showed ring enhancement. The SLD of our 68 patients was stationary in 51 patients (75%), while it was increased in 11 patients (16.2%) and decreased in the remaining 6 (8.8%) (Table 3).

The analysis of ring enhancement at first follow-up CT showed a significant but weaker relationship with overall survival (*p* = *0.016*) (Table 4). In contrast, the difference in enhancement between follow-up and baseline CT (*DELTA-RING*) was not significantly related to Overall Survival (OS).( Table 5).

**Table 4:** Kaplan–Meier analysis associating ring enhancement and overall survival at T1

Case processing summary				
Ring enhancement control	Total N.	N. of Events	Censored	
			N.	Percent (%)
No	38	38	0	0,0%
Yes	30	30	0	0,0%
Overall	68	68	0	0.0%

Means and medians for survival time								
Ringenhancement control	Mean^a^				Median			
	Estimate	Std. Error	95% Confidence interval		Estimate	Std. Error	95% Confidence Interval	
			Lower Bound	Upper Bound			Lower Bound	Upper Bound
No	17,544	,608	16,352	18,737	18,660	,272	18,127	19,193
Yes	11,476	1,046	9,425	13,527	11,530	,199	11,140	11,920
Overall	14,867	,677	13,539	16,195	17,280	2,077	13,209	21,351

Overall comparisons			
	Chi-Square	df	Sig
Log Rank (Mantel-Cox)	5,779	1	,016

a. Estimation is limited to the largest survival time if it is censored,

Test of equality of survival distributions for the different levels of ring.enhanc.controllo.

**Table 5:** DELTA RING associated with OS

Case processing summary				
Delta ring	Total N.	N. of Events	Censored	
			N.	Percent (%)
Decrease	6	6	0	0,0%
Same	51	51	0	0,0%
Increase	11	11	0	0,0%
Overall	68	68	0	0,0%

Means and medians for survival time								
Delta ring	Mean^a^				Median			
	Estimate	Std. Error	95% Confidence Interval		Estimate	Std. Error	95% Confidence Interval	
			Lower Bound	Upper Bound			Lower Bound	Upper Bound
Decrease	16,453	2113	12,313	20,594	17,870	2,661	12,554	23,086
Same	15,051	,704	13,670	16,431	17,280	2,312	12,748	21,812
Increase	13,151	2,417	8,413	17,889	10,610	,058	10,495	10,725
Overall	14,867	,677	13,539	16,195	17,280	2,077	13,209	21,351

Overall comparisons			
	Chi-Square	df	Sig
Log Rank (Mantel-Cox)	,776	2	,678

a Estimation is limited to the largest survival time if it is censored

Test of equality of survival distributions for the different levels of delta.ring.

After excluding several correlated features, SHAPE_STAGING_Sphericity, and GLCM_STAGING_Contrast were considered the most significant features related to ring enhancement and then to build the binary logistic model (*p* = *0.025* and *p* = *0.077*, respectively).

**Fig. 3:**
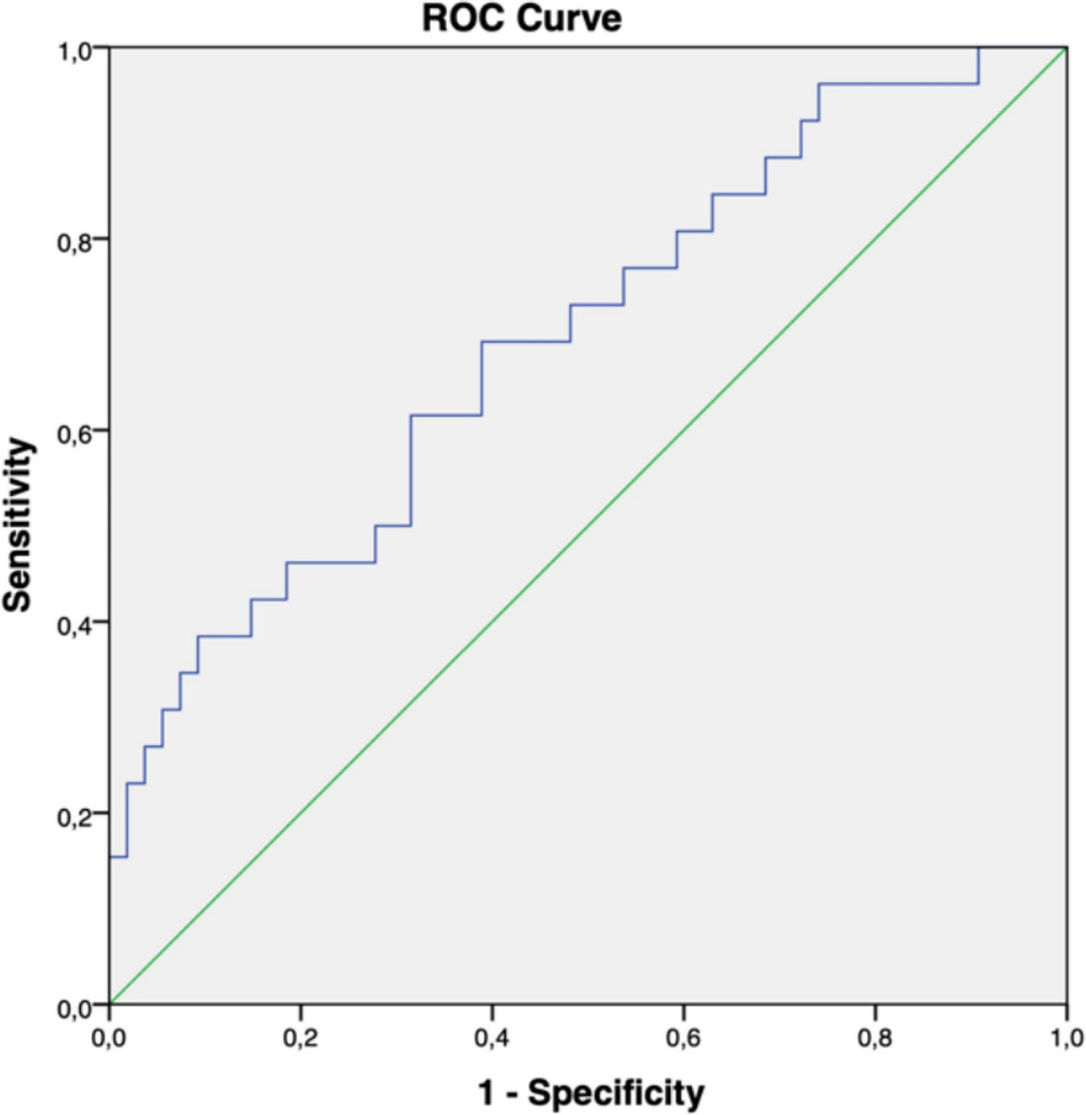
Performance of the radiomic model correlated to ring enhancement

The model performed an AUC of 0.695, where SHAPE_STAGING Sphericity was significantly related to ring enhancement (*p* = *0.003*) (Fig. 3).

## Discussion

In this retrospective cohort of bevacizumab-treated patients with CRLM, we found that a visually appreciable portal-phase rim (ring) enhancement on contrast-enhanced CT was consistently associated with inferior overall survival (OS), both at baseline and at the first follow-up. This qualitative association held when assessed at baseline and on early follow-up imaging. By contrast, simple quantitative density (HU) changes between baseline and first follow-up did not reach statistical significance for outcome prediction in our series, suggesting that the prognostic signal of ring enhancement is primarily phenotypic/qualitative rather than purely densitometric. To move beyond human-eye assessment, we extracted radiomic features and found that the shape descriptor “sphericity” (SHAPE_STAGING Sphericity) correlated with the ring-enhancement phenotype and, in turn, with prognosis.

Lower sphericity reflects irregular margins and heterogeneous peripheral perfusion, features that are known to correspond to biologically aggressive tumor subtypes and relative resistance to anti-angiogenic therapy. This provides a plausible mechanistic explanation for the association observed in our cohort.

We now highlight that the integration of a simple, interpretable radiologic sign with quantitative radiomics adds incremental value beyond size-based metrics.

Our observations align with and extend published evidence that peripheral (rim) enhancement on the portal venous phase reflects viable tumor at the lesion–liver interface and portends inferior treatment response and shorter progression-free survival in CRLM, including in patients receiving bevacizumab-based chemotherapy [24, 26]. In a dedicated MultiDetector Computed Tomography (MDCT) study, portal-phase enhanced rims independently predicted 1-year progression-free survival (PFS) and poor response to bevacizumab-containing regimens [24]. Histopathology-correlated work further shows that lesions with an enhanced rim harbor more viable cells and less infarct-like necrosis, reinforcing the biological plausibility that a pronounced rim signals aggressive biology and relative treatment resistance [26]. These data are consistent with a broader body of imaging science indicating that anti-angiogenic effects may not be captured adequately by size or attenuation alone, and that morphologic criteria outperform RECIST for prognostication in bevacizumab-treated CRLM [32].

Radiomics offers a quantitative framework to capture tumor heterogeneity and microenvironmental patterns that are not visible at the naked eye [33, 34]. Seminal overviews established the rationale and key pitfalls of high-throughput feature extraction and modeling, and the field has matured with standardized feature definitions and reproducibility initiatives [15]. Focused on CRLM, recent studies report prognostic radiomics signatures from CE-CT that predict disease-free survival (DFS)/recurrence and survival, often complementing or outperforming clinical risk scores and size-based metrics and, in some cases, achieving external validation [35, 36]. Our finding that a shape feature (sphericity) relates to outcome is in keeping with contemporary analyses in which shape belongs among the most informative domains and where original shape sphericity has emerged as a prognostic signal for overall survival [21].

Systematic syntheses of radiomics in liver metastases—including CRLM-enriched cohorts—converge on a consistent pattern: at baseline, higher entropy and lower homogeneity are associated with better survival and higher chemotherapy response rates [21, 37]; after effective treatment, entropy tends to fall and homogeneity rises, paralleling tumor regression grade and improved outcomes [38]. Similar dynamics are reported for skewness (lower baseline values and post-therapy increases associated with response) [21]. While this may seem counterintuitive, a plausible interpretation is that pre-treatment heterogeneity partly reflects perfused, angiogenic viable rim amenable to anti-angiogenic/chemotherapy effects, whereas highly uniform lesions at baseline may represent tightly packed cells or necrosis, both linked to poorer chemosensitivity [21]. In our study, first-order and GLCM features did not retain independent association with the qualitative rim phenotype or outcomes after correlation filtering—an observation that mirrors known challenges in feature redundancy and instability across acquisition settings and underscores the value of explainable anchors [21].

A reliable, non-invasive biomarker that anticipates benefit from bevacizumab-based regimens remains a clinical priority. Recent multimodal models (e.g., PET/CT plus clinical data) and CT/MRI radiomics pipelines show encouraging performance for predicting bevacizumab efficacy and pathologic response, supporting the feasibility of imaging-based treatment selection [39]. By coupling a human-interpretable phenotype (portal-phase rim) with quantitative shape metrics, our work contributes an interpretable and operational framework that could be tested prospectively as a triage tool.

In the era of new technologies and minimally invasive liver surgery (MILS) [40, 41], also in peripheral centers as demonstrated in Hub and Spoke learning program [42, 43], patients who respond to induction therapy may achieve conversion to resection with excellent perioperative outcomes, while those with limited oligometastatic burden who progress on systemic therapy may be candidates for thermal ablation (radiofrequency or microwave) as part of a multimodality plan [44]. High-level data support these principles: intensive chemotherapy (e.g., FOLFOXIRI + bevacizumab) increases response and conversion rates [45]; and the international COLLISION phase III trial suggests non-inferiority of thermal ablation vs resection for ≤ 3 cm resectable CRLM, emphasizing the role of precise risk stratification to allocate patients to surgery vs ablation [46].

Recent literature has increasingly emphasized that the surgical indication for CRLM should be guided not only by anatomical feasibility but also by the biological and radiologic response to systemic therapy—particularly when antiangiogenic agents such as bevacizumab are employed. Several studies have demonstrated that response to bevacizumab-based chemotherapy represents a pivotal determinant for surgical selection, resectability, and postoperative outcomes [45, 47].

A pooled analysis of prospective studies by Cremolini et al. [45] and more recent long-term data by Dong et al. [47] confirmed that FOLFOXIRI + bevacizumab regimens achieve high conversion-to-resection rates and improved overall survival in patients with initially unresectable, liver-limited disease. The magnitude of morphologic and radiologic response—particularly the disappearance of peripheral enhancement and the development of homogeneous hypoattenuation—correlates strongly with histopathologic tumor regression and increased likelihood of R0 resection. These findings support an early reassessment for surgery in patients showing partial but biologically favorable response to bevacizumab-containing therapy.

Conversely, non-responding lesions—identified by persistent ring enhancement, irregular contours, or lack of necrotic transformation—are associated with poor pathologic response and limited benefit from continued systemic treatment [24, 26]. Such imaging features may guide a shift toward alternative and this approach aligns with the currents recommendations advocating a dynamic, biology-driven reassessment of resectability during systemic therapy [22].

Emerging data from multidisciplinary cohorts suggest that morphologic responders to bevacizumab experience improved long-term survival compared with non-responders [32]. Moreover, radiomics-based studies such as Zhou et al. [39] have shown that quantitative imaging features can predict bevacizumab efficacy and identify patients most likely to benefit from surgery following anti-VEGF therapy, providing a non-invasive decision-support framework for multidisciplinary teams.

Collectively, these observations substantiate the concept that the response to bevacizumab-based chemotherapy should serve as a decisive clinical and biological criterion for surgical eligibility. Patients achieving morphologic or radiomic response may safely proceed to resection even in the absence of major dimensional reduction, while early surgery may be considered in non-responders to avoid disease progression during futile systemic therapy. This evidence consolidates the integration of response-guided, biologically adaptive surgical strategies into current CRLM management algorithms, harmonizing with the precision oncology paradigm endorsed by modern guidelines.

Persistent ring enhancement—despite stable lesion size—may indicate suboptimal response and should prompt reconsideration of surgical timing within multidisciplinary evaluation, whereas responders might be more appropriate candidates for conversion resection.

So, thanking into account the consideration above, our pilot study may contribute to better define patient specific characteristics concerning tumor progression, and consequently impact on indication for liver resection.

The growing integration of digital postoperative monitoring tools further supports the shift toward minimally invasive and patient-centered follow-up strategies [48], especially in the era of telemedicine [49], aligning with radiomics-based, non-invasive evaluation models. In this context, interpretable imaging biomarkers and radiomic analytics can inform personalized management strategies for CRLM [50].

The lack of prognostic significance for absolute density changes over a short interval in our series is concordant with evidence that anti-angiogenic effects remodel lesion interface and parenchymal/tumor architecture more than they produce early, robust HU shifts. Morphologic CT criteria—rather than size or HU alone—better align with pathologic regression and survival in bevacizumab-treated CRLM [32].

## Limitations

The limitations of the study include the retrospective design; single-center workflow; modest sample size and early attrition; unmasked imaging assessment; potential scanner/protocol heterogeneity (slice thickness, reconstruction kernel) that can affect radiomic stability; and lack of external validation. These are well-recognized impediments to radiomics translation and motivate IBSI-compliant pipelines, rigorous feature reproducibility checks, and multicenter validation. Moreover, we did not incorporate histopathological growth patterns or molecular markers (e.g., RAS/BRAF, MSI), which are emerging covariates with independent prognostic relevance and may interact with imaging phenotypes.

## Conclusions

In this retrospective cohort of patients with colorectal liver metastases treated with bevacizumab-based chemotherapy, a visually appreciable portal-phase rim (ring) enhancement on contrast-enhanced CT was consistently associated with inferior overall survival, both at baseline assessment and on the first follow-up scan, whereas short-interval changes in attenuation (HU) failed to show prognostic significance.

Beyond this qualitative phenotype, a complementary quantitative signal emerged: the shape descriptor sphericity correlated with ring enhancement and contributed to a modestly discriminative model, supporting the concept that interpretable radiologic features and parsimonious radiomics can be combined to capture biologically relevant tumor behavior.

Taken together, these findings argue for incorporating portal-phase rim enhancement, supplemented by shape-based metrics, into early imaging response assessments for CRLM under anti-angiogenic therapy, where size and densitometry alone may be insufficient. If prospectively validated across centers and harmonized acquisition protocols, this biomarker pair could refine risk stratification and treatment selection within multidisciplinary pathways—informing the timing of conversion surgery and the allocation to locoregional therapies when appropriate—while maintaining clinical interpretability.

Emerging evidence suggests that morphologic and radiomic responses to bevacizumab-based chemotherapy constitute key biological and clinical criteria for surgical selection. Favorable responders may safely undergo hepatic resection even without major size reduction, reflecting the vascular normalization and cytoreductive effects of anti-VEGF therapy. Conversely, early surgery may be warranted in non-responders to prevent disease progression. These insights support response-guided, biologically adaptive surgical strategies that align with the precision oncology framework of modern CRLM management.

Given the single-center, retrospective design and limited sample size, these results should be considered hypothesis-generating and warrant external validation with standardized, IBSI-compliant pipelines and integration of molecular and histopathologic covariates. Nevertheless, the convergence of a readily appreciable CT sign with a robust, explainable radiomic feature provides a pragmatic foundation for imaging biomarkers that are both clinically usable and biologically plausible in the management of CRLM.

## Data Availability

The datasets generated or analyzed during the study are available from the corresponding author on reasonable request.
